# Damage signals in the insect immune response

**DOI:** 10.3389/fpls.2014.00342

**Published:** 2014-07-11

**Authors:** Robert Krautz, Badrul Arefin, Ulrich Theopold

**Affiliations:** Department of Molecular Biosciences, Wenner-Gren Institute, Stockholm University, StockholmSweden

**Keywords:** coagulation, innate immunity, tumor, hemocytes, nematodes, danger

## Abstract

Insects and mammals share an ancient innate immune system comprising both humoral and cellular responses. The insect immune system consists of the fat body, which secretes effector molecules into the hemolymph and several classes of hemocytes, which reside in the hemolymph and of protective border epithelia. Key features of wound- and immune responses are shared between insect and mammalian immune systems including the mode of activation by commonly shared microbial (non-self) patterns and the recognition of these patterns by dedicated receptors. It is unclear how metazoan parasites in insects, which lack these shared motifs, are recognized. Research in recent years has demonstrated that during entry into the insect host, many eukaryotic pathogens leave traces that alert potential hosts of the damage they have aﬄicted. In accordance with terminology used in the mammalian immune systems, these signals have been dubbed danger- or damage-associated signals. Damage signals are necessary byproducts generated during entering hosts either by mechanical or proteolytic damage. Here, we briefly review the current stage of knowledge on how wound closure and wound healing during mechanical damage is regulated and how damage-related signals contribute to these processes. We also discuss how sensors of proteolytic activity induce insect innate immune responses. Strikingly damage-associated signals are also released from cells that have aberrant growth, including tumor cells. These signals may induce apoptosis in the damaged cells, the recruitment of immune cells to the aberrant tissue and even activate humoral responses. Thus, this ensures the removal of aberrant cells and compensatory proliferation to replace lost tissue. Several of these pathways may have been co-opted from wound healing and developmental processes.

## INTRODUCTION

Insects have served as excellent tools to study different aspects of innate immunity. Initially the focus in insect immunology was on the identification of microbially activated pathways and effector mechanisms. During recent years, the contribution of wound signals and of signals associated with the damage that occurs during the infection process has become increasingly appreciated. Despite the significant conceptual overlap between “Danger” and “Damage,” we will refer to them as “Damage-induced signals” or “damage” (Matzinger, 2002). The fruit fly *Drosophila melanogaster* has been particularly useful to map the underlying pathways. Using the fly as a model also provides insight into the relation between immunity and other aspects of the animal’s physiology and normal as well as aberrant development.

## *Drosophila* AS A TOOL TO STUDY INSECT IMMUNITY

The innate immune system in insects comprises two central and several peripheral tissues. The central tissues are (1) the fat body, which combines the tasks of the vertebrate liver and the adipose tissue and (2) different types of blood cells, which are collectively called hemocytes. The main function of the fat body within the immune system is to release soluble factors into the hemolymph (Lemaitre and Hoffmann, 2007). Some of these factors are produced constitutively others only after immune stimulation. Secreted proteins are antimicrobial peptides but also factors that are required during clot formation after wounding (see below). Induction of their transcription is achieved via the Toll and imd pathways which are located downstream of recognition molecules that bind microbial elicitors such as peptidoglycan and beta-1,3 glucan (Lemaitre and Hoffmann, 2007).

In addition to their involvement during wound healing, which is discussed below, insect hemocytes have several functions (Williams, 2007; Krzemien et al., 2011); (1) they phagocytose smaller foreign intruders such as bacteria (2) they form capsules around larger intruders such as wasp eggs and (3) they form extracellular aggregates (nodules) as a defense against larger numbers of small intruders,.

Both encapsulation and nodule formation lead ultimately to melanization due to the activity of the enzyme phenoloxidase. The zymogen prophenoloxidase is released from specialized hemocytes and activated in the hemolymph via a proteolytic cascade (Cerenius et al., 2008).

*Drosophila* hemocytes include three cell types (Williams, 2007): (1) plasmatocytes which are the largest fraction in the hemolymph, perform phagocytosis and release immune effector and signaling molecules, (2) crystal cells, which contain prophenoloxidase in a crystalline form and other proteases of the prophenoloxidase-activating system (PAS; Bidla et al., 2005, 2007) and (3) lamellocytes, which are rare in naïve animals but differentiate and participate during encapsulation. Interestingly lamellocytes are also produced upon sterile wounding (Markus et al., 2005) indicating that their production can be triggered by damage-associated signals. In wounds, activated crystal cells rupture, leading to the massive release of cytosolic material including the PAS. Regulation of this process includes the TNF homolog Eiger and may also depend on damage signals (Bidla et al., 2007), possibly the cleavage of Eiger by metalloproteinases similar to the situation in tumors (see below).

Peripheral tissues comprising for example the tracheae, the epidermis, the gonads and the gut epithelium rely on the more locally restricted release of effectors such as prophenoloxidase and antimicrobial peptides and on the production of reactive oxygen species to varying extent. The induction of immune reactions in these tissues depends primarily on the imd pathway (Davis and Engstrom, 2012).

## HISTOLOGICAL STUDIES OF WOUND HEALING

Early histological studies of wound healing in insects were primarily performed on model insects that were easy to raise under laboratory conditions such as the mealworm *Tenebrio mollitor,* the blood-sucking bug *Rhodnius prolixus* and the wax-moth *Galleria mellonella* (Rowley and Ratcliffe, 1978; Theopold et al., 2004). Many of these insect species have additional classes of hemocytes and unfortunately the nomenclature is far from consistent. Lepidopteran insects have two major classes of blood cells, namely granulocytes and plasmatocytes (Lavine and Strand, 2002). As the name implies, granulocytes contain various types of vesicles, which are differentially released upon activation. They are also able of phagocytosis. Lepidopteran plasmatocytes have a similar function as their *Drosophila* counterpart during encapsulation but contain a more homogenous cytoplasm. They also perform the task of *Drosophila* lamellocytes, which are not present in lepidopterans (Lavine and Strand, 2002). The crystal cell equivalents in lepidopterans are called oenocytoids, which are larger in size and contain a homogenous cytosol (Gupta, 1984; Lavine and Strand, 2002). The highly variable composition of hemocyte types amongst insect species reflects an adaption to their respective environment and its specific pathogens. Thus, the prevalence of a particular set of immune cell types appears as an ecological trade-off indicating the necessity to allocate resources to the dominant immune challenges. As such, the constitution of the immune system can also provide clues as to which pathogens are encountered by the species of interest in its natural environment.

*Galleria* wound healing was followed during 72 h after wounding in a detailed histological study (Rowley and Ratcliffe, 1978) and shown to include several stages: initially, a plug formed, which included fat body fragments, and hemolymph. Hemocytes were also part of the initial plug. Most of these were granular cells, which had degranulated and subsequently degenerated and a few plasmatocytes. The plug had started to melanize after 60 min due to the release and subsequent activation of the enzyme prophenoloxidase from oenocytoids. Farther away from the initial plug, a loose network of hemocytes that had formed earlier condensed into a more compact layer, which included both degranulated granular cells, plasmatocytes, and oenocytoids as well as the hemolymph clot which acted as a universal glue. Further melanization and additional recruitment of blood cells was at later time points followed by the migration of epidermal cells across the wound site, which by 24 h had formed a continuous layer and started to secrete a new cuticle. The formation of a new epidermis and cuticle continued during later time points (Rowley and Ratcliffe, 1978). It was found that at the cellular and histological levels, wound closure bears many similarities with the formation of capsules and nodules all of which recruit the same cell types in the same order and ultimately lead to melanization (Ratcliffe and Rowley, 1979).

Wound healing in *Drosophila* larvae occurs in a similar way as in *Galleria* although, since the cell counts are lower in fly larvae, fewer cells become part of the clot. Nevertheless a similar melanized scab forms within an hour after wounding larvae (Galko and Krasnow, 2004). The scab also activates epidermal cells, which close the wounds and form a syncytium (Losick et al., 2013). Re-epithelialization is established by movement of epithelial cells and regulated via the JNK pathway, which forms a gradient that peaks both at the wound site and a few cell layers away from it (Rämet et al., 2002; Galko and Krasnow, 2004; Lesch et al., 2010). The importance of phenoloxidase during *Drosophila* wound healing was shown in mutants that lack crystal cells. These larvae produced diffuse scabs and most died within 24 h after wounding (Rämet et al., 2002; Galko and Krasnow, 2004; Lesch et al., 2007). Formation of a scab and JNK-activation could be physiologically separated by using a different mode of wounding, which involves pinching larvae without creating open wounds (Galko and Krasnow, 2004). Under such circumstances neither clotting nor melanization occurred but most of the epithelial responses were still observed. Further genetic analysis showed that in the absence of crystal cells, the JNK-pathway was hyper-induced. Taken together, this indicated that wound healing at the histological level was quite similar between *Galleria* and *Drosophila* although the relative infiltration of hemocytes into the wound site appears to differ. This may explain the different pattern of melanization in these two species and be due to different modes of activation (see below).

Despite the similarities in wound healing across insect orders, there are also evolutionary differences indicating again the adaption to specific immune stimuli. When clots were prepared *ex vivo* from a comprehensive array of insects, the histological appearance and the types of cells involved showed a great deal of variability for example in some species, cells appeared to lyze during the process leading to their separate classification as coagulocytes while at the other extreme blood cells appeared dispensable for clotting in some other species (Gregoire, 1974).

## MOLECULAR ANALYSIS OF THE *Drosophila* CLOT

As evident from the study by Galko and Krasnow (2004) mentioned above, *Drosophila* offers a great variety of tools for molecular analysis. This was further enhanced through the early availability of the whole genome sequence for the fly in 2000 (Gregoire, 1974). Other methodological advances important for the study of *Drosophila* immunity include the response to wounding. Wounding is part of most artificial immunization protocols (Lemaitre and Hoffmann, 2007), which were used to study insect immunity but wounding epithelia is also a necessary step of gaining entry into the host for many natural pathogens.

Methods particularly suitable to study wounding include:

• Different wounding regimes such as poking with different sized needles, pinch wounds and laser-induced wounding, which allowed live imaging (Galko and Krasnow, 2004; Stramer et al., 2005).
• Molecular markers for hemocytes such as antibodies with specificity for different subclasses of blood cells as well as reporter lines with hemocyte-specific markers, which can be used *in vitro* and *in vivo* (Kurucz et al., 2007; Csordas et al., 2014).
• An increase in available mutant lines that were created and mapped more rapidly based on the available genome data and the development of novel transposable elements used for mutagenesis (Thibault et al., 2004; Venken et al., 2011).
• Epidermal driver lines, which allow following wound healing *in vivo* and in real time as well as reporters with specificity for the sub-cellular components and the signaling pathways involved (Lesch et al., 2010).
• An increasing number of natural infection models which involve breaches to epithelial barriers (Vodovar et al., 2005; Cronin et al., 2009; Arefin et al., 2014).

The availability of the genome sequence permitted proteomics approaches to isolate components of the clot that forms during scab formation (Karlsson et al., 2004; Scherfer et al., 2004). Both cellular and humoral components in the hemolymph were found to contribute to clot formation. Cellular factors include phenoloxidase (Bidla et al., 2005), hemolectin, which contains several domains that also occur in mammalian clotting factors (Goto et al., 2003; Lesch et al., 2007) and possibly transglutaminase (Johansson et al., 2005) although this enzyme may be provided from other sources as well. Humoral factors include lipophorin, some hexamerins and Fondue (Karlsson et al., 2004; Scherfer et al., 2004; Scherfer et al., 2006). Functional analysis showed that despite defects in several *in vitro* assays for clotting efficiency, mutants or knockdown lines specific for clotting factors showed subtle bleeding defects and only a slight reduction in mortality (Lesch et al., 2007; Lindgren et al., 2008; Chang et al., 2012). This may be due to redundancy in clotting similar to humans, where bleeding defects of different severities are observed (Chang et al., 2012). Strikingly some clotting mutants had immune defects instead indicating that clotting serves a more specific immune function (Hyrsl et al., 2011; Chang et al., 2012). In particular knockdown of transglutaminase led to an increased susceptibility to insect pathogenic nematodes (Wang et al., 2010). A protective function for clots in this infection model was corroborated in further studies on mutants in fondue, one of the structural clot components (Hyrsl et al., 2011). Since transglutaminase, which crosslinks proteins via lysine and glutamine residues is homologous to the mammalian clotting factor XIIIa, plasma from patients who lack factor XIIIa was studied and shown to be far less capable of sequestering bacteria than plasma from healthy individuals (Wang et al., 2010). A protective role for factor XIIIa during septic infections was further confirmed in a mouse model. Therefore the immune function of the clot appears to be conserved during evolution (Loof et al., 2011a,b). Not surprisingly in the light of its immune function, the clot is a target for a virulence factor that is produced by a nematode with specificity for *Drosophila* (Toubarro et al., 2013).

The analysis of mutants in prospective immune genes led to a rapid increase in our understanding of the molecular organization of the signal transduction pathways in the immune system (Lemaitre and Hoffmann, 2007; Wang et al., 2013). Much of these results confirmed the importance of microbial patterns and their recognition during the activation of effector mechanisms. Both the *Toll* and *imd* pathway were shown to be activated through recognition of microbial elicitors in particular peptidoglycan and fungal elicitors such as beta 1,3-glucans (Lemaitre and Hoffmann, 2007; Wang et al., 2013; **Figure 2**). Either downstream cleavage of proteases or direct recognition by surface molecules on immune tissues triggers the immune response. Nevertheless parallel to the microbially activated branch of the *Toll* path, a second activation path was discovered, which fitted well with the notion that the invasive activity of the microbial intruder directly activates immunity: persephone, one of the amplification enzymes in the *Toll* pathway was shown to be a direct substrate for a fungal proteases (PR1) that is essential for breaching the cuticular barrier (**Figure 2**; Gottar et al., 2006). All this occurs extracellularly but bears striking similarity with the concept behind the guard hypothesis, which explains the activation of plant immunity through the traces that virulence factors leave on effector-induced plant immune mechanisms, while manipulating them (De Wit et al., 2009). The difference is that virulence factors leave their traces within plant cells whereas the cleavage of Persephone acts as a damage sensor in the hemolymph, which informs the insect about the presence of an intruder by way of its proteolytic activity (Gottar et al., 2006). And just like peptidoglycan is indispensable for the bacterial life cycle, so is proteolytic activity for a pathogen that relies on breaching host barriers. In fact it has recently been proposed that the same mechanism acts as an m-sensing module in apoptosis-deficient flies (Ming et al., 2014). In addition, the multistep-cascades leading to the activation of the *Toll*-pathway as well as the ultimate cleavage of the prophenoloxidase might not only be interpreted as a highly regulated safety mechanism preventing their uncontrolled activation (Tang, 2009). In line with the above characterized mechanism, both proteinase cascades can also be seen as a platform for sensing proteolytic activity in the hemolymph that either refers to states of altered self or infection. The former is accompanied by the release of proteases during various forms of controlled and uncontrolled cell death as shown for instance for caspase-1 in the crayfish model (Jearaphunt et al., 2014). The complex serine protease cascades are equally well able to accommodate more, yet unidentified proteases of further pathogens. Furthermore, serpins as inhibitory regulators of the serine protease cascades can account for the systemic containment of the signal. Hence, the balance between activating signals and serpins establishes a localized signal gradient, which can also serve to recruit hemocytes to sites of tissue alteration or inflicted wounds (Bidla et al., 2009).

## DIFFERENT WAYS TO HURT A FLY

++- To gain further insight into re-epithelialization after wounding without interference from the scab Lesch et al. (2010) further developed the pinch wound assay by adding epidermal drivers that facilitated visualization of this process. After confirming the influence of JNK signaling, candidate genes that are part of JNK signaling and genes involved in cytoskeletal remodeling were tested. Knockdowns showed different phenotypes and led to a more detailed picture of epithelial cell migration where cells adjacent to the wounds migrated and closed the wound and cells farther away contributed to wound closure through elongation (Lesch et al., 2010). Wounding through laser ablation in embryos demonstrated that cells adjacent to the wound extend filopodia-like extension which help cells from opposite ends of the wound to re-establish cellular contacts and involves a purse string mechanism where actin cables form a transcellular network that contracts to close the wound (Wood et al., 2002). Hydrogen peroxide released from embryonic wounds attracts hemocytes similar to what had been observed in zebrafish (Niethammer et al., 2009; Moreira et al., 2010). Production of hydrogen peroxide depends on the NADPH oxidase DUOX (Moreira et al., 2010). Hydrogen peroxide synthesis is in turn activated through a calcium flash followed by a wave of calcium release from the injured tissue (Razzell et al., 2013). The presence of hemocyte specifically expressed genes such as phospholipase A2 and the homolog of a mouse inflammatory gene (GADD45) at the wound site provides further support to the idea that the response against damage signals at wound sites has conserved features (Stramer et al., 2008). Notably the phospholipase has a protective function in an infection model that involves wounding (Hyrsl et al., 2011). In contrast to larvae, wounding of embryos does not result in the formation of a hemolymph clot (Stramer et al., 2005). Further screens after embryonic wounding complete the picture of the cytoskeletal changes that occur during wound closure and healing (Abreu-Blanco et al., 2011, 2012, 2014). In larvae, where clotting occurs in open wounds hemocytes appear to be directly captured into the wound site from circulating hemolymph without evidence for a requirement of chemo-attractive cues at least when pinch wounds are applied (Babcock et al., 2008). The initial attachment is to cellular debris and may be mediated by some unidentified damage signals. In contrast to open wounds, the hemocytes in pinch wounds are released back into circulation after the epidermis has healed. In parallel to JNK signaling, wound healing in epidermal cells involves the receptor tyrosine kinase Pvr (the *Drosophila* PDGF/VEGF homolog) and its ligand Pvf1. Damage in this case appears to be signaled by exposure of Pvr at the wound edges which attracts Pvf1 and activation of the formation of the cellular processes that close the wounds (Wu et al., 2009). The parallel activation of the JNK pathway leads to the dedifferentiation of cells at the wound margin (Wu et al., 2009). Additional receptor tyrosine kinases required during wound healing are the epidermal growth factor receptor (EGFR) homolog (Geiger et al., 2011) and the Ret-family receptor Stitcher (Tsarouhas et al., 2014). Stit acts in a dual way, which contributes on the one hand to the formation of the actin-ring and to re-epithelialization and on the other hand to the activation of transcriptional responses via the transcription factor grainy head (grh; Wang et al., 2009; Tsarouhas et al., 2014). The two modes of activation require interaction of Stitcher with Src-like kinases or the transducer protein Drk (downstream of receptor kinase), respectively (Tsarouhas et al., 2014).

**FIGURE 1:**
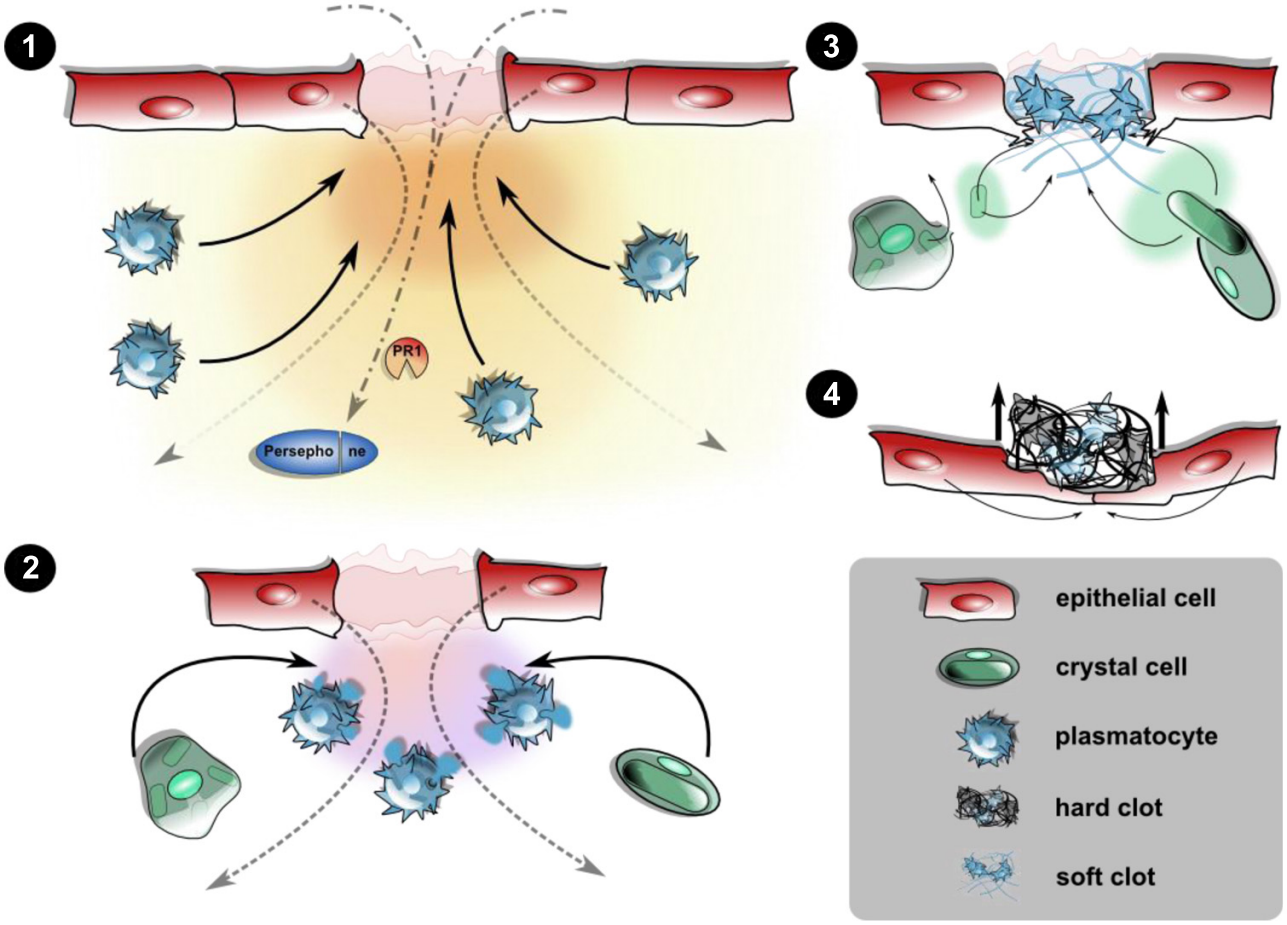
**Cellular and humoral responses upon epithelial wounding.** Disruption of the epidermis and the larval cuticle (omitted for simplicity) initiates an immediate response prior to wound healing leading to the closure of the wound site. **(1)** Wound signals attract hemocytes: the destructed epithelial layer releases yet unidentified damage signals creating a gradient along which plasmatocytes are recruited toward the wound site. This may be actively including the release of hemocytes from the sessile compartment or passive capture of hemocytes from the hemolymph. Wound sites serve as entry ports for pathogens too, leading to activation of PRRs and damage receptors like Persephone. **(2)** Degranulation and soft clot formation: at the wound site, plasmatocytes degranulate initiating the formation of a soft clot involving humoral factors from the hemolymph, too. Crystal cells are incorporated at this stage. **(3)** Melanization and hard clot formation: the rupture of crystal cells releases prophenoloxidase, which after activation of the PAS melanizes the soft clot. Degranulated plasmatocytes as well as the ruptured remnants of the crystal cells are incorporated into the clot matrix. **(4)** Re-epithelization and shedding of the fully formed hard clot (scab): the formation of protruding filopodia precedes the closure of the epithelium, leaving the scab expelled during the course of re-epithelialization. Wound closure is aided by changes in the cytoskeletal architecture and formation of a syncytium (see text for further details).

Several natural infection models also include wounding as part of the infection process including parasitoid wasps and entomopathogenic nematodes (Schmidt et al., 2001; Wertheim et al., 2005; Hallem et al., 2007; Arefin et al., 2014). Most notably, during infection of mosquitoes with *Plasmodium*, ookinetes invade the hemolymph via the gut epithelium (Vega-Rodriguez et al., 2014). Damage signals upon Plasmodium infection of mosquitoes are discussed in detail in the article by Moreno-Garcia et al. in this issue. Entomopathogenic nematodes enter their hosts either via the cuticle or the gut epithelium (Eleftherianos et al., 2010). In both routes, the epithelial tissues are damaged mechanically due to the activity of the nematodes’ specialized mouth organs. So far no microbial patterns that are particular to the nematode have been discovered, therefore the induction of the immediate response against nematodes may be triggered by the damage aﬄicted to the host or by microbial elicitors derived from the nematodes’ symbiotic bacteria (Castillo et al., 2013). Nematode infection of *Drosophila* larvae and adults has been established during past years and used to study the ensuing immune response (Hallem et al., 2007; Dobes et al., 2012; Castillo et al., 2013). Classification of the genes that are differentially regulated during nematode infections identified many that are induced in other infection models (Arefin et al., 2014). Notably additional genes in non-immune paths belong to pathways that have been implicated in wound healing and regenerative processes in other model species (Arefin et al., 2014). For two genes that belong to immune classes, *in vivo* evidence for an immune function had previously been lacking but could be confirmed using nematode infection. Similarly, knockdown lines for a component of the basement membrane showed increased mortality upon nematode infection (Arefin et al., 2014). Interestingly, further histological analysis of the wounds after transmigration demonstrated that collagen IV, another component of the basement membrane had been lost in the closer vicinity of the wounds, which had melanized after nematode invasion of the hemolymph (Arefin et al., 2014; **Figure 3**). The loss of collagen from the wound site is most likely due to the activity of metalloproteinases, which are known virulence factors for entomopathogenic nematodes (Cabral et al., 2004). Collagen fragments produced by the activity of microbial metalloproteinases have indeed been shown to act as damage signals in *Galleria* (Altincicek and Vilcinskas, 2006). The underlying scenario appears to resemble the one described above for the protein Persephone (see **Figure 1**), which also acts as a detector for a proteolytic activity that is indispensable for host invasion (Altincicek et al., 2009). Additional peptides that are produced upon exposure to the protease thermolysine and have immune-stimulatory activity have been isolated from *Galleria* hemolymph and may also act as damage signals (Berisha et al., 2013).

**FIGURE 2:**
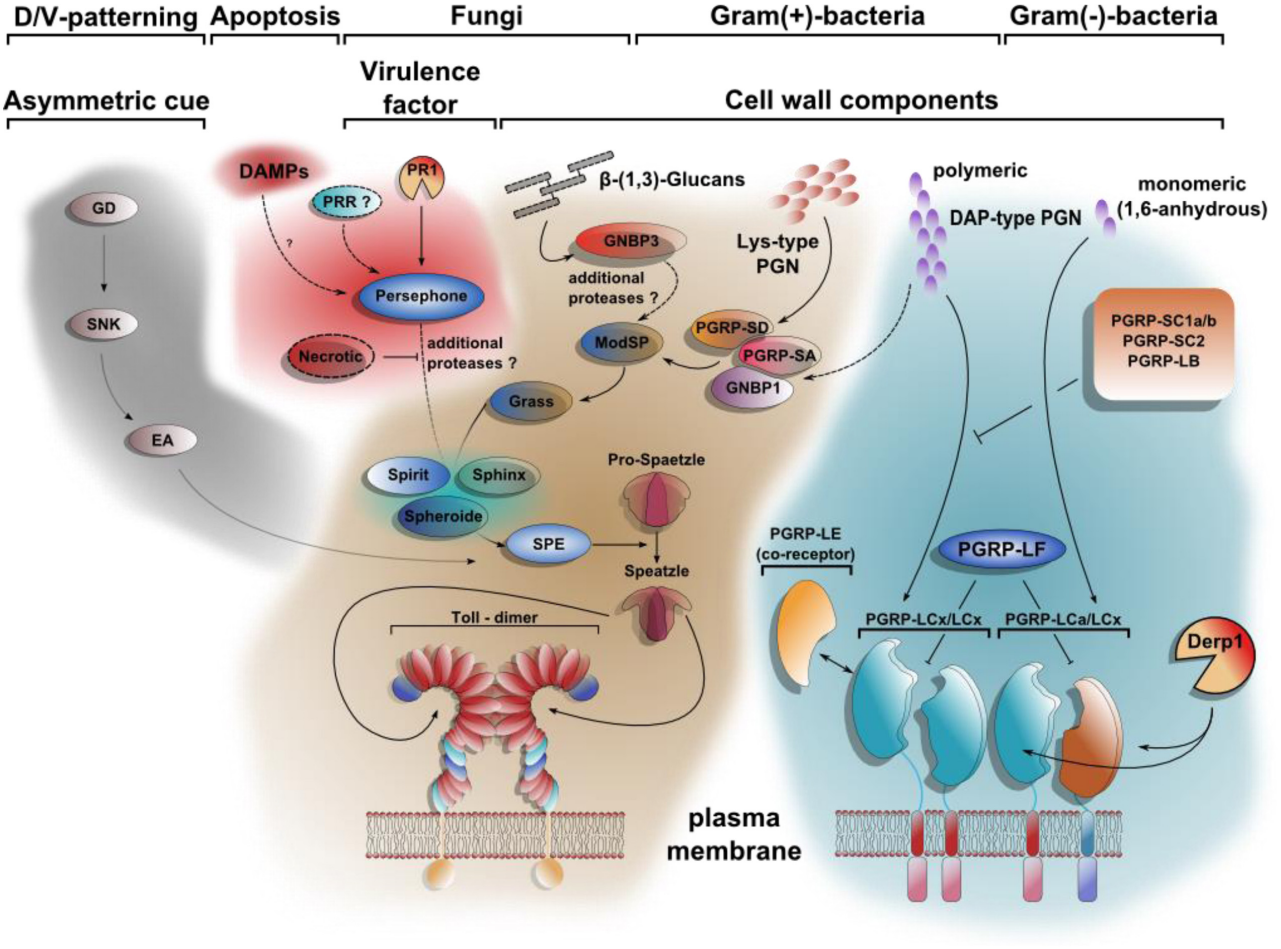
**Toll signaling integrates DAMPs and PAMPs.** At least four different extracellular pathways activate Spätzle proteolytically. The first comprises a cascade of serine proteases, which defines dorso-ventral polarity in the developing egg. Gastrulation defective (GD), Snake (SNK), and Easter (Ea) induce the hydrolytic cleavage of Spätzle on the ventral side of the embryo. DAMPs can be sensed by Persephone (Psh) as shown for a model with defects in apoptosis. The proteolytic cleavage of Psh by the fungal protease PR1 renders it active as well. The pattern recognition receptor GNBP3 (Gram negative bacteria binding protein 3) senses β-1,3 glucan-stretches from fungal cell walls parallel to PAMPs. The Toll pathway is also required for resistance toward Gram-positive bacteria due to the recognition of polymeric Lys-type PGN by PGRP-SA, PGRP-SD, and GNBP1. Whereas the downstream signaling of the Toll-activating pattern recognition receptors (PRRs) converge on the modular serine protease (ModSP), the Persephone-mediated Damage-signal will be integrated by the secreted Sphinx1/2-, Spheroide- and Spirit-serine proteases, resulting in the activation of the Spätzle-activating enzyme (SPE). In contrast to the extracellular multistep activation cascade of the Toll-pathway, the stimulation of Imd-signaling is achieved by direct binding of PGN to receptor dimers. PGRP-LE, PGRP-LF, PGRP-SC1a/b, PGRP-SC2, and PGRP-LB all share regulatory functions. Similar to Psh-activation by PR1-hydrolysis, PGRP-LC can be activated after cleavage by Der p 1 [modified after (Ferrandon et al., 2007; Lemaitre and Hoffmann, 2007)].

**FIGURE 3:**
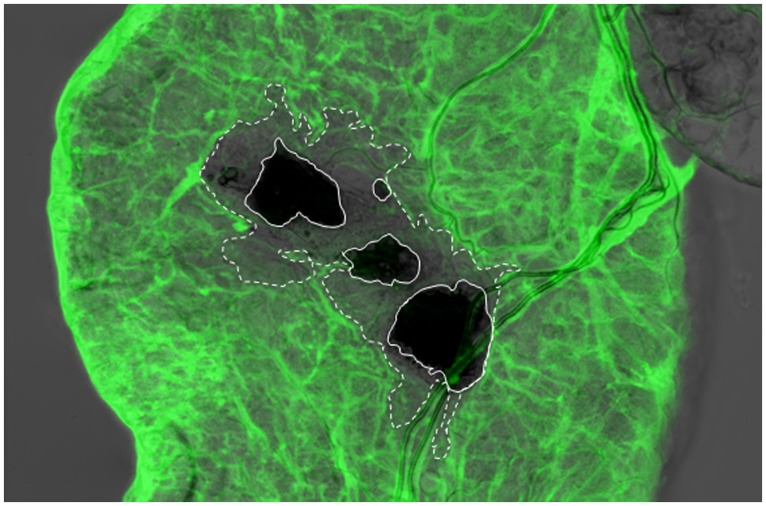
**Integrity of the basement membrane (BM) is disrupted around the wound site upon nematode infection of the *Drosophila* larva.** Viking (Collagen IV)-GFP fusion protein (protein trap) was used as a basement membrane reporter to analyze nematode infected *Drosophila* gut using confocal microscopy. Expression of the Viking-GFP is controlled by the endogenous promoter and enhancer elements. Note that Viking-GFP (green) signal was missing beyond the melanized area in the wound site (dark black, straight line, the area that lacks the green signal is outlined by the dashed line), which was caused by the nematode (Arefin et al., 2014; re-produced with kind permission from S. Karger AG, Basel).

## AN ORGANISM VIEW OF WOUNDING

One of the key concepts resulting from the early studies on *Toll* signaling in *Drosophila* was the cooption of developmental pathways for immune functions. Similarly it has been pointed out that wound closure shows similarities at the cellular level with the process of dorsal closure during embryonic development (Wood et al., 2002). Co-opting the same genes or signal transduction modules for different tasks bears the risk of mutual competition and it has indeed been found that hemocytes do respond to conflicting signals that guide on the one hand their migration during embryonic development and on the other hand attract them to laser-induced wounds. The developmental stage, the time that has passed after wounding and the distance from the wound site collectively influence the distribution of embryonic hemocytes (Moreira et al., 2010). In larvae wounding appears to lead to a systemic wound response that involves the central, nervous system. This signal is induced by large wounds and depends on alternative activation of the PAS via a newly discovered hemolymph protease (Hayan; Nam et al., 2012). The redox dependent signal activates the JNK pathways in nerve cells, which activate a cytoprotective program in the whole organism. Crosstalk between wounded cells and the central nervous system after UV irradiation can also modify the behavior of larvae, and lead to an increased response to noxious thermal stimuli and to a response to previously non-noxious stimuli. Strikingly, the communication between harmed apoptotic cells and nociceptive neurons in the epidermis is mediated by the *Drosophila* equivalent of tumor necrosis factor (Eiger) and its receptor (Wengen), which may act again as a damage receptor as well as Hedgehog signaling (Babcock et al., 2009, 2011). Additionally, the interaction between the nervous system and the hemocytes is mutual. In the hematopoietic pockets of the larval segments, hemocytes form clusters intertwined with sensory neurons. The latter transmit signals to the residing, sessile hemocytes, which might even elicit immune responses in case of noxious stimuli (Makhijani et al., 2011; Makhijani and Bruckner, 2012).

Finally the histological changes that have been observed after wounding *Galleria* and *Drosophila* have become amenable to genetic analysis. RacGTPase activity could be shown to be required for formation of a syncytium from the cells adjacent to the wound site and Hippo signaling via the effector Yorkie for the same process and for polyploidization of cells near the wound site (Losick et al., 2013).

## SIMILARITIES BETWEEN WOUNDS AND TUMORS: MESSAGES FROM THE FLY

Tumor mutants were among the earliest fly mutants identified in nature (surveyed in Gateff, 1978). Both over- and invasive growth of the mutant tissues could be observed and subsequent molecular analysis identified both genes that had been implied in tumor growth in other organisms including humans as well as novel candidate tumor genes (Kounatidis and Ligoxygakis, 2012). During the past 15 years, tumor growth in flies has been induced experimentally often in a mosaic fashion for example in the mitotically active imaginal discs, which are precursors of adult organs. Tumors were most often induced with a combination of a dominant-active form of the oncogene *Ras* and reduced expression of genes that define cell polarity (Brumby and Richardson, 2003; Pagliarini and Xu, 2003; Kounatidis and Ligoxygakis, 2012; Stefanatos and Vidal, 2012). Induction of mutant clones for example in the eye imaginal disc and simultaneous expression of a GFP-marker allowed measuring the tumor size in different genetic backgrounds (Uhlirova and Bohmann, 2006). In addition, invasive growth of the mutant tissue into the neighboring central nervous tissue was observed and could also be quantified. This led to the identification of several pathways that modify tumor growth and invasiveness including the JNK, TNF (Eiger) and JAK/STAT pathways (Kounatidis and Ligoxygakis, 2012). Similarities between wounds and tumor clones were observed, both of which expressed metalloproteinases, showed degradation of the adjacent basement membrane and attracted hemocytes (Pastor-Pareja et al., 2008). The TNF pathway plays a dual role during tumor control: cells adjacent to tumor clones activate TNF-signaling to drive tumor cells into apoptosis thus limiting tumor growth (Igaki et al., 2009). Therefore TNF appears to also act as a damage-activated sensor. One model is that surface-bound Eiger becomes available as a ligand through the activation of metalloproteinases. In contrast, in hemocytes where TNF is also active, the downstream expression of metalloproteinases promotes tumor progression (Cordero et al., 2010). It appears therefore that hemocytes can have both tumor-limiting as well as tumor-promoting activity similar to macrophages where the dividing line between the two effects appears to coincide with the M1/M2 macrophage distinction (Allavena et al., 2008).

Even sole expression of active Ras has been found to lead to cellular dysplasia (Christofi and Apidianakis, 2013; Hauling et al., 2014). In the midgut this induced compensatory proliferation of stem cells, while in the hindgut, cells detach form the epithelia and delaminate into the hemolymph (Jiang and Edgar, 2011; Jiang et al., 2011; Bangi et al., 2012; Christofi and Apidianakis, 2013). Expression of active Ras in the salivary glands (using the Beadex driver) induces apoptosis and expression of metalloproteinases and attracts hemocytes. Concomitantly an immune response is activated in the fat body including a set of genes that are activated by microbial intruders as well as a set that is specifically induced in the presence of overgrowing tissue (Hauling et al., 2014). Amongst the induced genes shared by this model and wasp egg infestation a receptor for the import of retinoid precursors into cells (Santa-maria) was significantly upregulated (Wertheim et al., 2005). When apoptosis alone is induced with the Beadex driver the resulting signal has been shown to involve retinoids, which suppress ecdysone release and thus development. The resulting extended larval period allows the larvae to heal the damage due to apoptosis and similarly, the damage after gamma-irradiation (Halme et al., 2010). Conversely although upon expression of active Ras, the larval period is extended, too, the resulting pupae never eclose (Hauling et al., 2014). This fits with the idea that tumor tissues show features of wounds but – in contrast to wounds – never heal (Dvorak, 1986).

## DAMAGE AND THE LOSS OF CELLULAR HOMEOSTASIS

Of course not all tumors and not all stages of cancer progression are regarded as wounds. It has been debated which markers distinguish an immunogenic from a non-immunogenic form of tumor (Guo et al., 2014). Tumor-associated markers as well as damage signals are obvious candidates as elicitors of tumor-specific responses (Guo et al., 2014). A key point as to whether a response is activated is how much the mutated cell manages to cope with its aberration by activating endogenous control mechanisms (Campisi and d’Adda di Fagagna, 2007; Kroemer et al., 2013). Primarily, these control mechanisms will arrest the cell cycle such as via the action of p53 or lead to an adjustment of cellular metabolism for example by activation of autophagy (Kroemer et al., 2013; Guo et al., 2014). In case the endogenous mechanisms fail, apoptosis may be activated, which is mostly regarded as a non-inflammatory form of cellular demise (Vicencio et al., 2008). An alternative is the induction of a senescent phenotype, which leaves the cell metabolically active but prevents further divisions. While the above-mentioned cellular pathways leave the cell intact, they often lead to the dislocation of cellular components, which can be perceived as a damage signal (Vicencio et al., 2008; Kroemer et al., 2013). Examples include the exposure of the inner membrane lipid phosphatidyl serine on the surface of apoptotic cells and the release of calcineurin and ATP and high mobility group antigen from damaged cells (Peter et al., 2010; Kroemer et al., 2013). Ultimately, cells may be driven into necrosis, which is usually regarded as a strong inflammatory stimulus. In *Drosophila* wounds, hemocytes have been found to express apoptotic markers followed by induction of necrosis (Bidla et al., 2007). The full physiological consequences remain to be investigated but the expression of apoptotic phosphatidylserine (PS) is also a common marker for activated of platelets in mammalian blood clots (summarized in Theopold et al., 2002). In fly larvae PS has been shown to activate the PAS independent of exogenous signals (Bidla et al., 2009). Therefore PS qualifies as a bona fide phylogenetically conserved damage signal across animals. Similarly, epidermal DNA damage activates the PAS as well as other immune responses (Karpac et al., 2011). In addition to the more abundant classes of hemocytes that may eventually die a necrotic death in the clot, some specialized classes of hemocytes such as *Drosophila* crystal cells rupture within minutes after bleeding releasing the components of the PAS (Bidla et al., 2007). This is different from the activation of neutrophils, which leads to the release of NETs (Brinkmann et al., 2004) since crystal cell nuclei stay intact (Krautz et al., unpublished data). Nevertheless, this ultimate mode of secretion is expected to lead to a massive release of intracellular components, many of which may act as damage signals.

One damage-associated signal that activates phagocytosis in *Drosophila* hemocytes is the above-mentioned calcineurin (Asgari et al., 2003). Interestingly, a calcineurin-like protein is also expressed in the venom of an endoparasitoid wasp, which may therefore interfere with the activation via damage signals (Zhang et al., 2006).

## ALLERGIES AND DAMAGE

Despite their bad reputation allergies can also be regarded as deregulated responses against noxious environmental substances such as venoms, xenobiotics, irritants and haematophagous fluids (Palm et al., 2012) many of which are introduced via wounds. The responses are normally beneficial and may include strengthening barrier defenses or the expulsion, inactivation or sequestration of noxious substances (Palm et al., 2012). Many of these reactions are also an option for insects, for example during formation of the clot and during gut inflammation (Wang et al., 2010; Buchon et al., 2013). Since the harmful agents are very diverse and often abiotic, it has been proposed that they induce immune reaction due to their damaging effects on cells and tissues (Palm et al., 2012). Examples include the major dust mite antigen Der p 1, which is a protease and phospholipases from bee venom. This is reminiscent of the sensing mechanisms that involve microbial proteases, which lead to cleavage of Persephone or Collagen. Strikingly, in *Drosophila* the mite protease Der p 1 cleaves the surface receptor PGRP-LC leading to activation of the *imd* pathway, providing yet another example for the key role of damage signals in insect immunity (Warmbold et al., 2013). PGRP-LC cleavage also activates melanization (Schmidt et al., 2008), one of the insect responses with the largest potential for detrimental side effects which is therefore tightly regulated (Eleftherianos and Revenis, 2011). It may be premature to draw parallels between allergies in mammals and insect responses but it is increasingly recognized that – similar to allergic reactions – immune responses in insects generally can have serious side effects which can be more extensive than the damage created by the infectious agent itself (Dionne et al., 2006; Shirasu-Hiza and Schneider, 2007). The regulatory pathways that limit this damage are only starting to be understood.

## AUTHOR CONTRIBUTIONS

Ulrich Theopold wrote the initial draft of the manuscript, all authors contributed to the manuscript. Robert Krautz designed and drew **Figures 1** and **2**, Badrul Arefin drew **Figure 3**. All authors discussed the content of the manuscript.

## Conflict of Interest Statement

The authors declare that the research was conducted in the absence of any commercial or financial relationships that could be construed as a potential conflict of interest.

## ABBREVIATIONS

GNBP: Gram-negative binding protein
JAK/STAT: Janus kinase and signal transducer and activator of transcription
JNK: c-Jun N-terminal kinase
NETs: neutrophil extracelluar traps
PAS: prophenoloxidase-activating system
PDGF: platelet-derived growth factor
PGRP: peptidoglycan recognition protein
PRR: pattern recognition receptor
PS: phosphatidylserine
VEGF: vascular-endothelial growth factor.
